# Evaluation of DNA Variants Associated with Androgenetic Alopecia and Their Potential to Predict Male Pattern Baldness

**DOI:** 10.1371/journal.pone.0127852

**Published:** 2015-05-22

**Authors:** Magdalena Marcińska, Ewelina Pośpiech, Sarah Abidi, Jeppe Dyrberg Andersen, Margreet van den Berge, Ángel Carracedo, Mayra Eduardoff, Anna Marczakiewicz-Lustig, Niels Morling, Titia Sijen, Małgorzata Skowron, Jens Söchtig, Denise Syndercombe-Court, Natalie Weiler, The EUROFORGEN-NoE Consortium, Peter M. Schneider, David Ballard, Claus Børsting, Walther Parson, Chris Phillips, Wojciech Branicki

**Affiliations:** 1 Institute of Forensic Research, Section of Forensic Genetics, Krakow, Poland; 2 Department of Genetics and Evolution, Jagiellonian University, Krakow, Poland; 3 Faculty of Life Sciences, King’s College, London, United Kingdom; 4 Section of Forensic Genetics, Department of Forensic Medicine, Faculty of Health and Medical Sciences, University of Copenhagen, Denmark; 5 Department of Human Biological Traces, Netherlands Forensic Institute, The Hague, The Netherlands; 6 Forensic Genetics Unit, Institute of Forensic Medicine, Faculty of Medicine, University of Santiago de Compostela, Santiago de Compostela, Spain; 7 Genomic Medicine Group, Centro de Investigación Biomédica en Red de Enfermedades Raras (CIBERER), Institute of Health Carlos III, Madrid, Spain; 8 Institute of Legal Medicine, Medical University of Innsbruck, Innsbruck, Austria; 9 Department of Analytical Biochemistry, Jagiellonian University Medical College, Krakow, Poland; 10 Department of Dermatology, Medical College of Jagiellonian University, Krakow, Poland; 11 Institute of Legal Medicine, Medical Faculty, University of Cologne, Cologne, Germany; 12 Forensic Science Program, The Pennsylvania State University, University Park, Pennsylvania, United States of America; National Cancer Institute, National Institutes of Health, UNITED STATES

## Abstract

Androgenetic alopecia, known in men as male pattern baldness (MPB), is a very conspicuous condition that is particularly frequent among European men and thus contributes markedly to variation in physical appearance traits amongst Europeans. Recent studies have revealed multiple genes and polymorphisms to be associated with susceptibility to MPB. In this study, 50 candidate SNPs for androgenetic alopecia were analyzed in order to verify their potential to predict MPB. Significant associations were confirmed for 29 SNPs from chromosomes X, 1, 5, 7, 18 and 20. A simple 5-SNP prediction model and an extended 20-SNP model were developed based on a discovery panel of 305 males from various European populations fitting one of two distinct phenotype categories. The first category consisted of men below 50 years of age with significant baldness and the second; men aged 50 years or older lacking baldness. The simple model comprised the five best predictors: rs5919324 near *AR*, rs1998076 in the 20p11 region, rs929626 in *EBF1*, rs12565727 in *TARDBP* and rs756853 in *HDAC9*. The extended prediction model added 15 SNPs from five genomic regions that improved overall prevalence-adjusted predictive accuracy measured by area under the receiver characteristic operating curve (AUC). Both models were evaluated for predictive accuracy using a test set of 300 males reflecting the general European population. Applying a 65% probability threshold, high prediction sensitivity of 87.1% but low specificity of 42.4% was obtained in men aged <50 years. In men aged ≥50, prediction sensitivity was slightly lower at 67.7% while specificity reached 90%. Overall, the AUC=0.761 calculated for men at or above 50 years of age indicates these SNPs offer considerable potential for the application of genetic tests to predict MPB patterns, adding a highly informative predictive system to the emerging field of forensic analysis of externally visible characteristics.

## Introduction

Androgenetic alopecia (AGA), often termed male pattern baldness (MPB), is a widespread type of gradual hair loss from the scalp that is particularly frequent among European males. The etiology of male pattern baldness has been widely researched and although it is still not fully understood, major factors behind this condition have been identified to comprise genetic predisposition and hormone dependency [1]. It has been estimated that approximately 80% of the variance of AGA-related hair loss can be explained by genetic factors [2,3]. Multiple studies including several recent genome-wide association analyses have disclosed a large number of single nucleotide polymorphisms (SNPs) associated with AGA susceptibility and provided strong evidence that the region on chromosome X, which includes the two neighboring genes of androgen receptor (AR) and ectodysplasin A2 receptor *(EDA2R)*, is involved in MPB [4–12]. Although involvement of the Xq12 region in AGA appears to be undisputed, various reports are discordant in terms of the number of loci and effect size associated with particular variants located near the *AR/EDA2R* genes. Fine mapping of this region by Brockschmidt et al. in 2010 indicated locus rs12558842, located upstream of *AR* and *EDA2R*, has the strongest association with AGA and suggested there are likely to be 1–2 causative variants in this region regulating expression of *AR* and/or *EDA2R* genes [10]. The complexity of genetic determination of MPB was further highlighted by a study that reported three SNPs from the *AR/EDA2R* region to be monomorphic among Asians, concluding that this locus is unlikely to play any role in AGA in Asia [13]. A region located at 20p11 represents the second MPB susceptibility locus, discovered independently by two genome wide association studies (GWAS) and further confirmed in both Europeans and Asians. Effect size attributed to this locus was found to be lower than *AR/EDA2R* indicating approximately two-fold increased risk of AGA in Europeans and Asians [9,13,14]. GWAS followed by fine-mapping analysis has confirmed the significance of both chromosome X and chromosome 20 regions [10], whereas a further study by the same group in 2011 identified *HDAC9* in 7p21.1 to be a novel susceptibility locus for AGA [15]. The large meta-analysis confirmed the association of Xq12, 20p11, and *HDAC9* while discovering five additional loci associated with early onset baldness in Europeans. These novel AGA risk loci comprised: region 17q21.31; *TARDBP* (chr1); *HDAC4* (chr2); *AUTS2* (chr7); and *SETBP1* (chr18) [12]. In the follow-up study, Heilmann et al. (2013) investigated 12 genomic regions for which Li et al. (2012) found suggestive associations, however these additional regions did not achieve genome-wide significance [16]. Nevertheless, Heilmann’s study revealed association with MPB for four additional regions: 2q35 (*WNT10A*); chr3q25 (*SUCNR1*); chr5q33.3 (*EBF1*); and chr12p12.1 (*SSPN*), extending the list of known AGA susceptibility loci to 12 potential predictors for this condition.

Genetic association is often a first step to discover the biological function of genetic variants [17]. The identified DNA variants may also find direct application in prediction modeling, which in medical genetics can be used to estimate the risk to develop a disease. In forensic DNA analysis such markers can be applied to predict externally visible characteristics (EVCs) of an unknown person, whose DNA is available for analysis [18,19]. Male hair distribution is one of the most obvious externally visible traits and thus the prediction of MPB will be useful in forensic investigations. Prediction modeling performed on other EVCs has shown that the current known DNA markers associated with iris pigmentation are sufficient to predict blue and brown eye colour [20], while hair colour [21] and chronological age [22–24] have recently been added as informative tests. Other appearance traits like stature or facial morphology are much more complex and need further extensive studies before they can be added to predictive frameworks in forensic analysis [25,26]. In the present study, we investigated the loci that have been associated with androgenetic alopecia with the aim to evaluate their significance for MPB and assess their potential as predictors for early onset baldness in European males.

## Materials and Methods

### Ethics statement

Written informed consent was obtained from all sample donors. The study was approved by each regional bioethics committee: the Commission on Bioethics of the Regional Board of Medical Doctors in Krakow (48 KBL/OIL/2008); the Danish committee system on biomedical research ethics (H-3-2012-023); the KCL BDM Research Ethics Subcommittee (BDM/13/14-111); the USC ethics committee of clinical investigation in Galicia, Spain (CEIC: 2009/246) and followed approved internal procedures in the Netherlands Forensic Institute in The Hague.

### Sample collection

A total of 605 samples were collected from healthy male donors from the following European populations: 448 males from Poland, 49 from the Netherlands, 36 from the United Kingdom, 27 from Denmark, 20 from Italy, 13 from Germany and 12 from Spain. Phenotype classification involved direct inspection of subjects by a dermatology specialist or evaluation of multiple high quality photographs per subject. The degree of baldness was assessed on a seven-point scale according to the Norwood-Hamilton classification system [1,27]. The phenotypic descriptions of Norwood-Hamilton baldness categories are given in S1 Table. Collected samples were further divided into four phenotype categories distinguishing two extremes according to age/baldness severity: *phenotype category 1*, men below 50 years of age with significant baldness (Norwood-Hamilton grade III or higher; 226 samples), *phenotype category 2*, men aged 50 or older lacking baldness (Norwood-Hamilton grade I or II; 179 samples). The remaining subjects were divided into: *phenotype category 3*, men below 50 with no signs of baldness (Norwood-Hamilton grade I or II; 100 samples) and *phenotype category 4*, men aged 50 years or older with significant baldness (Norwood-Hamilton grade III or higher; 100 samples). Primary statistical analyses, including association testing and development of suitable prediction models, used 305 samples from extreme phenotype categories 1 (176 men) and 2 (129 men). This set included samples from Poland (73.4%), the Netherlands (12.5%), the United Kingdom (7.2%), Italy and Spain (6.9%). Such approach identified high-risk MPB alleles. The testing set of 300 independent samples was used to evaluate the model accuracy. These additional samples comprised of subset 1, 50 samples of *phenotype category 1;* subset 2, 50 samples of *category 2*; subset 3, 100 samples of *category 3* and subset 4, 100 samples of *category 4*. Initially, only the phenotypic extreme subsets 1 and 2 were used to evaluate predictive accuracy. This initial test set was about 30% of the discovery set with samples from Poland (59%), Denmark (10%), Germany (10%), the Netherlands (9%), the United Kingdom (6%) and Italy or Spain (6%). The developed prediction models were then further tested by expanding test sets with categories 3 and 4. This allowed assessment of the model’s performance in a general population to gauge usefulness when an unknown sample is examined. Discovery and testing sets were matched by age and degree of baldness. Details of the study sets used for association testing and subsequently for development and testing of prediction models are given in S2 and S3 Tables.

### Genotyping

A set of 45 SNPs was selected from previous AGA association studies, including all GWAS analyses [8,9,11,12,14–16,28,29]. An additional five hair morphology-associated SNPs were included in order to assess a recent hypothesis claiming a possible association between hair distribution and morphology [16,30]. Details of all selected candidate polymorphisms this study analyzed are given in S4 Table. DNA from the buccal swabs and whole blood was extracted using NucleoSpin Tissue extraction kit (Macherey-Nagel GmbH & Co.KG, Germany) [31], PrepFiler *Express* BTA DNA Extraction Kit or QIAamp DNA mini kit (Qiagen, Hilden, Germany). DNA was amplified in four PCR multiplex reactions consisting of 2.5 μL Qiagen Multiplex PCR mixture, 0.5 μL Q solution, 0.5 μl primer premix and 1.5 μL of 1–10 ng DNA. Thermocycling conditions were: 95°C for 15 min, 32 cycles of [94°Cx30s, 58°Cx90s, 72°Cx90s] then 72°C for 10 min. Most samples were genotyped using SNaPshot single base extension (SBE). PCR products were first purified using Exonuclease I (ExoI) and FastAP Thermosensitive Alkaline Phosphatase (FastAP) (Thermo Fisher Scientific, TFS), then SBE reactions used 1 μl of purified PCR product, 0.5 μl of SNaPshot mix, 0.5 of μl extension primer premix and DNase free water to 5 μl with 26 cycles of [96°Cx10s, 50°Cx5s, 60°Cx30s]. SBE products were purified with FastAP (TFS) and analysed with capillary electrophoresis using an ABI 3130xL Genetic Analyser. Further details are given in S5 Table. Samples from the UK were genotyped with next-generation sequencing using the KAPA Hyper Prep kit (Kapa Biosystems) according to manufacturer’s directions. Sequencing was made with the Illumina MiSeq system. SNP rs6945541 gave amplification problems due to duplication of the chr7 region where it is sited, necessitating one primer redesign and repeat genotyping. However, old and new primer sets gave 100% concordance (primer sequences in S5 Table).

### Statistical analyses

#### Population analysis

All variant data was analyzed for concordance with Hardy-Weinberg expectations using Arlequin v.3.1 (http://cmpg.unibe.ch/software/arlequin3). Haploview version 4.2 tested linkage disequilibrium between closely positioned SNPs (www.broadinstitute.org). Chromosome positions used in the linkage disequilibrium (LD) analysis were retrieved from GRCh37.p13 genome build (coordinates listed in S4 Table).

#### Association testing

The 50 SNPs were tested for association with MPB using binary logistic regression analyzed with IBM SPSS statistics v.22 (SPSS Inc., Chicago, IL, USA). The dependent variable was coded dichotomously as ‘1’ for *phenotype category 1* and ‘0’ for *phenotype category 2*. Independent variables (SNPs) were tested using additive (SNP genotypes coded 0, 1, 2 for the number of minor alleles); dominant (SNP genotypes coded ‘0’ for no minor allele or ‘1’ for at least one minor allele) and recessive (coded ‘0’ for no minor allele or one minor allele or ‘1’ for two minor alleles) inheritance modes. X-linked SNPs coded minor allele homozygotes as ‘2’ and major allele homozygotes as ‘0’. Odds ratios, corresponding 95% confidence intervals (CI) and p-values were estimated for all minor allele classifications. All analyses considered a P-value at <0.05 as statistically significant and correction for multiple hypothesis testing was not applied in this study. Minimal OR values detectable with a power of at least 80% were calculated with Power and Sample Size Program (PS Program) v.3.1.2 (http://biostat.mc.vanderbilt.edu/wiki/Main/PowerSampleSize).

#### Genotype risk score

As each tested SNP contributes to a small extent to individual risk of AGA, the combined effect of five SNPs selected by multivariate association analysis was assessed by estimating the Genotype Risk Score (GRS). GRS was calculated using a weighted risk alleles counting approach by adding up all weighted risk alleles (according to logistic regression β parameter) in five selected SNPs [12,32]. The resulting GRS was then divided into quartiles and the lowest quartile used as a reference to calculate the risk of AGA for the remaining three quartiles [12]. ORs and their respective p-values for designated quartiles were then assigned. The same procedure was applied to evaluate the combined effect of eight MPB loci described by Li et al. in their studied population set [12].

#### Epistasis testing

Analysis of SNP-SNP interactions was conducted on the discovery dataset using Multifactor Dimensionality Reduction (MDR v.2.0 beta 8.1, www.epistasis.org) and binary logistic regression (IBM SPSS statistics v.22) as previously described [31]. The MDR method identifies specific genotype combinations at ‘high risk’ for a given phenotype and uses an entropy-based approach to estimate the benefit in information gain from considering interactions between the tested SNPs [33–36]. Standard binary logistic regression was used to study pair-wise interactions at the multiplicative scale and for the disclosed epistatic effects ORs, respective 95% CIs and P-values were calculated.

#### Prediction modeling

Two prediction models were developed using logistic regression (IBM SPSS statistics v.22) applied to data from the discovery set of the 305 samples used for association and interaction analyses (S2 Table). The first prediction model included five SNPs selected as most significant using multivariate logistic regression. The second prediction model assessed twenty SNPs with positive, detectable impact on prediction accuracy. DNA variants increasing AUC by at least 0.001 were chosen. For this purpose, the ascertained SNPs were ranked according to their individual significance of association and sequentially tested for impact on prediction accuracy expressed by the area under the ROC curve (AUC). The AUC parameter indicates overall accuracy of prediction including sensitivity and specificity measured at different probability thresholds (an AUC value of 0.5 signifies complete lack of prediction and 1.0 perfect prediction). Those SNPs not showing statistical significance for association were also evaluated for their impact on prediction following the suggestions in a previous study [21]. Performance of the developed prediction models was evaluated with an independent test set by calculating AUC, sensitivity, specificity, positive predictive value (PPV), negative predictive value (NPV), total correct predictions for each phenotype category plus overall number of correct predictions [20,21,37,38]. Calculations were performed using 50% or 65% probability thresholds. Prediction results that failed to exceed the 65% probability threshold were considered inconclusive. The independent test set comprised 300 samples of the four age/phenotype categories that reflected the full range of MPB grade/age variation, making a general European male hair distribution ‘profile’ (S3 Table). Individuals from *phenotype categories 1* and *4* were treated as ‘bald’ and coded ‘1’ whereas *categories 2* and *3* were ‘non-bald’, coded ‘0’. Therefore, the ability of the developed prediction models to predict individuals in subsets 1 and 4 as ‘bald’ and subsets 2 and 3 as ‘non-bald’ was tested by their assignment to particular phenotype categories. The SNP-SNP interactions detected from MDR were also evaluated for their impact on prediction. The proportion of overall risk of AGA explained by the developed prediction models was estimated using the Nagelkerke pseudo-R^2^ statistic (IBM SPSS statistics v.22).

## Results

### Population data

Genotype data for 50 SNPs are provided in S6 and S7 Tables, for the discovery and testing sample sets respectively. The genotyping completion rate exceeded 99% for all markers. Analysis of the autosomal data in the full sample of 605 European men showed marginal deviation from Hardy-Weinberg equilibrium for two SNPs rs6137444 and rs1800547 (P>0.04, S8 Table). Details of LD analyses are shown in S1 Fig. Briefly, three haplotype blocks (r^2^>0.8) comprising 17 SNPs were found in the Xq12 region and one LD block comprising five SNPs (r^2^>0.8) was detected on chr20. Three SNPs from the chr17 region revealed the strongest LD (r^2^>0.9), while LD was also detected between rs2249817 and rs6461387 on chr7 (r^2^>0.8) and four SNPs on chr1 (r^2^>0.7).

### Association testing

**Fig 1:**
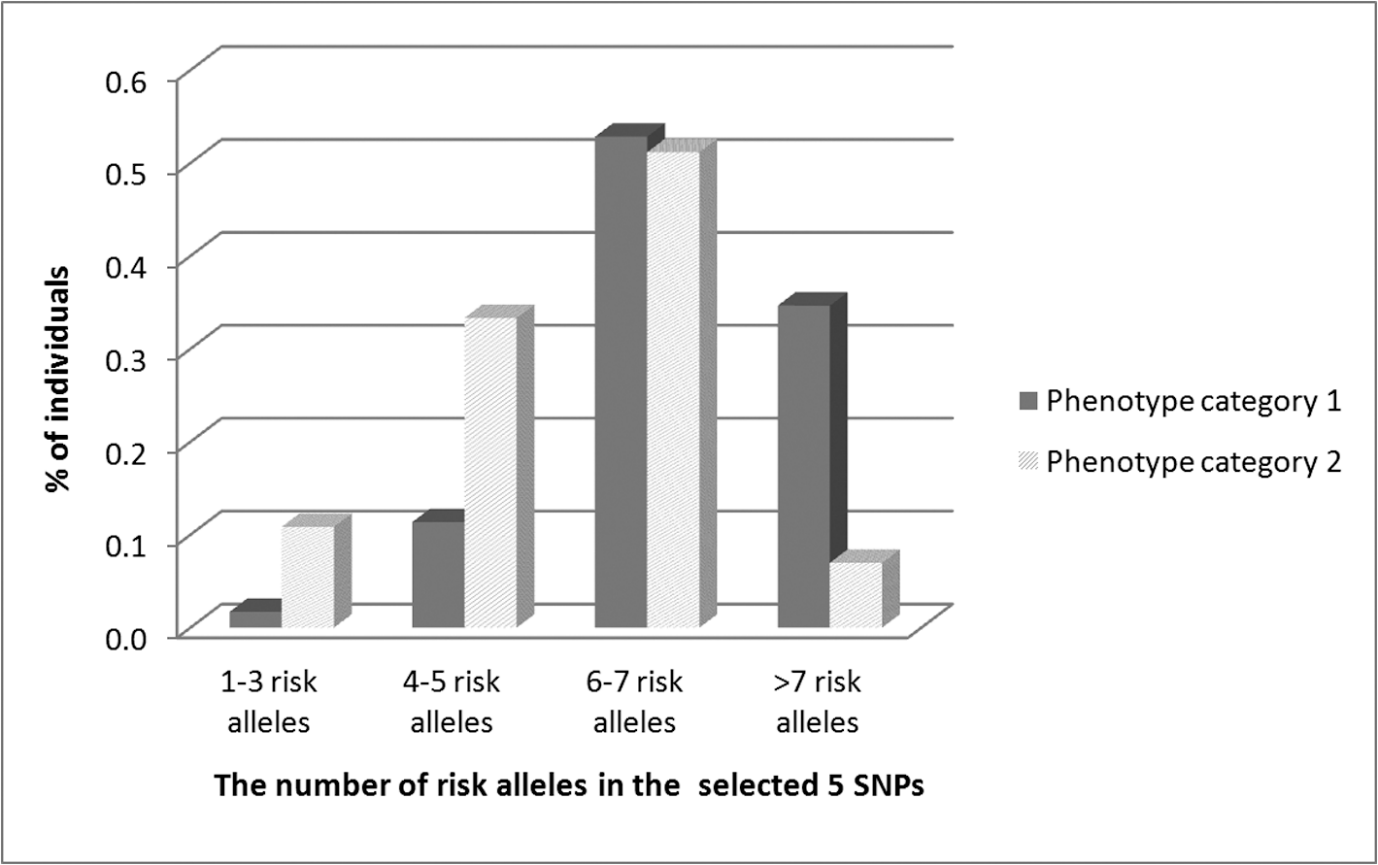
Numbers of risk alleles found in phenotype categories 1 and 2. Distribution of risk alleles in the top five SNPs selected by multivariate logistic regression for *phenotype category 1* (men <50 years with significant baldness) and men in *phenotype category 2* (≥ 50 years without baldness) of the discovery set samples.

From association testing of all 50 SNPs in 305 samples, 29 markers were found to be significantly associated with AGA in univariate analyses (S4 Table). Among the SNPs associated with MPB were 17 SNPs on Xq12, 8 SNPs on chr20, one SNP on chr1 (rs12565727), one SNP on chr5 (rs929626), one SNP on chr7 (rs756853) and one SNP on chr18 (rs10502861). The highest statistical significance was detected for rs5919324 positioned upstream of *AR* on Xq12 (OR = 0.536, 95% CI = 0.390–0.735, P = 1.119x10^-4^). From SNPs located on 20p11 the highest statistical significance was obtained for rs1998076 with OR = 0.541, 95% CI = 0.388–0.754 and P = 2.941x10^-4^. From the remaining chromosomes, high statistical significance was found for rs929626 (chr5) OR = 0.353, 95% CI = 0.205–0.608, P = 1.75x10^-4^; rs12565727 (chr1) OR = 0.478, 95% CI = 0.293–0.780, P = 3.11x10^-3^; and rs10502861 (chr18) OR = 0.531, 95% CI = 0.335–0.841, P = 7.0 x10^-3^. Notably, a higher significance was observed for these three SNPs when dominant allele categorization was applied. SNP rs756853 (chr7) was also significant with dominant allele categorization (OR = 1.760, 95% CI = 1.101–2.812, P = 0.018). The same association pattern in terms of the associated genes was observed for the entire discovery set and for a set restricted to Polish samples. Minor differences were observed in terms of association significance of various SNPs within the studied loci (data not shown). Multivariate association analysis considering the full 50 SNP set enabled selection of the most important polymorphisms showing independent effects in AGA (Table 1). The subsequent multivariate model included the five SNPs: rs5919324 (P = 9.883x10^-5^, OR = 0.480, 95% CI = 0.332–0.695); rs1998076 (P = 1.382x10^-4^, OR = 0.482, 95% CI = 0.331–0.701); rs929626 (P = 1.623x10^-3^, OR = 0.557, 95% CI = 0.387–0.801); rs12565727 (P = 2.591x10^-3^, OR = 0.493, 95% CI = 0.311–0.781) and rs756853 (P = 0.015, OR = 1.595, 95% CI = 1.094–2.324). Association testing confirmed the highest association for the known MPB susceptibility region on Xq12 containing *AR* and *EDA2R* loci. It was estimated that males with the rs5919324-T allele have 2.1 times increased risk to develop MPB compared to those with the C allele. It is notable that a similar increase of MPB risk was found to be associated with rs1998076-C on 20p11 (Table 1). The remaining three most significant SNPs associated with AGA susceptibility were rs12565727-T (chr1), rs929626-T (chr5) and rs756853-C (chr7) increasing the risk to develop baldness by factors of 2.0, 1.8 and 1.6 respectively. The combined effect of these five principal MPB predictors (indicated by multivariate logistic regression) was further evaluated by GRS estimation from division into quartiles. The risk score associated with the genotypes from the highest quartile vs. the lowest quartile was estimated to equal OR = 3.135 (95% CI = 2.242–4.386, P = 2.840x10^-11^), although an increased risk of MPB was detected in each quartile (Table 2). The mean number of risk alleles in the selected five top SNPs in the highest GRS quartile was 7.97. Analysis of all genotypes in the five SNPs in discovery set samples showed there was a significantly higher proportion of men from *phenotype category 1* possessing more than seven risk alleles compared with men from *phenotype category 2* (χ^2^ P = 1x10^-8^, Fig 1). It is worth highlighting that the combined effect of eight MPB loci identified by the study of Li et al. (2012) was actually lower (OR = 2.641).

**Table 1:** Results of multivariate logistic regression analysis for AGA performed on a discovery set consisting of 305 samples.

Multivariate logistic regression analysis							
Variables in model	Genes	Chr	Allelic variants	MAF	OR (95% CI)	Risk allele	P value of variable in model
rs5919324	*up of AR*	X	C/T	C (0.17)	0.480 (0.332–0.695)	T	9.833x10^-5^
rs1998076	*-*	20	C/T	T (0.39)	0.482 (0.331–0.701)	C	1.382x10^-4^
rs929626	*EBF1*	5	C/T	C (0.47)	0.557 (0.387–0.801)	T	1.623x10^-3^
rs12565727	*TARDBP*	1	C/T	C (0.18)	0.493 (0.311–0.781)	T	2.591x10^-3^
rs756853	*HDAC9*	7	C/T	C (0.39)	1.595 (1.094–2.324)	C	0.015

MAF: Minor allele frequency; OR: Odds ratio for the minor allele; CI: Confidence interval

**Table 2:** Genotype risk score associated with the risk of male pattern baldness.

Quartiles of a genotype risk score	OR (95% CI)	P value
Quartile 1	Reference	-
Quartile 2	2.717 (2.037–3.623)	1.079x10^-11^
Quartile 3	2.865 (2.114–3.891)	1.422x10^-11^
Quartile 4	3.135 (2.242–4.386)	2.840x10^-11^

### Prediction modeling

**Fig 2:**
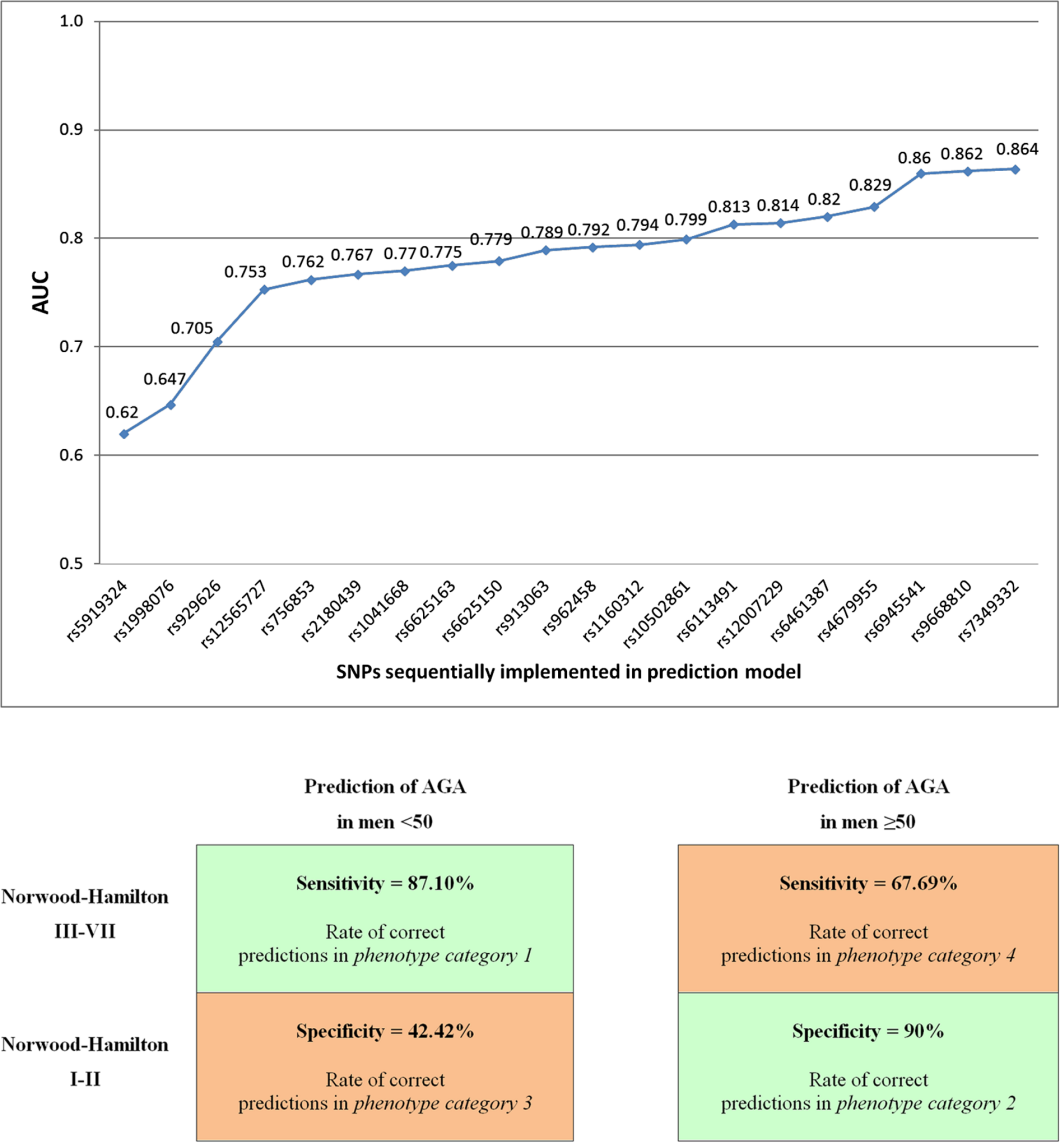
A. Contributions to the AUC value of twenty SNPs from the extended model of MPB prediction. B. AGA prediction parameters for the 20-SNP model. Rate of correct predictions in two groups of men, aged <50 and ≥50 years, and four distinct phenotype categories using a 65% probability threshold.

Two prediction models were developed based on genotype data obtained for the discovery set of 305 individuals from *phenotype categories 1* and *2*. The first model included the five most associated SNPs selected by multivariate association analysis. According to the Nagelkerke pseudo-R^2^ statistic, this model explained ∼22% of the total risk to develop MPB with the overall significance P = 1.772x10^-10^. This model was tested using an independent set of 300 males from all four phenotype categories. Initially, only the two subsets consisting of 50 samples each from *phenotype category 1* and *category 2* were used to test accuracy. These initial analyses indicated the simple MPB prediction model has an overall predictive accuracy expressed as AUC = 0.762. Applying the 50% probability threshold the overall number of correct predictions was 66% (66/100). However, the 65% probability threshold increased correct predictions to 75.8% (47/62), while 38 samples produced inconclusive results (S9 Table). Testing performed on the 300 sample set (with additional 100 samples each from *phenotype category 3* and *category 4*), reduced correct predictions to 54.7% and AUC = 0.594. Predictions for this dataset also remained weak with the 65% probability threshold, with 58.4% correct predictions (108/185) and 38.33% inconclusive results (S9 Table). This obvious drop of predictive accuracy was mainly caused by a near-complete lack of prediction success for *phenotype category 3*. The number of correct predictions of non-bald phenotype in this group was just 37.3% (S9 Table). Slightly lower predictive accuracy was also noted for *phenotype category 4* (≥50 years with significant baldness), where the level of correct predictions for baldness reached 60.9%. Therefore, good predictive performance was obtained for *categories 1* and *2* (87.1% and 71% correct predictions respectively using 65% probability threshold, S9 Table). The next stage of extended variant sets comprised 20 SNPs with positive impact on AUC: six in region Xq12 (rs5919324, rs1041668, rs6625163, rs6625150, rs962458, rs12007229), five in 20p11 (rs1998076, rs2180439, rs913063, rs1160312, rs6113491), three chr7 SNPs (rs756853, rs6461387, rs6945541) and singletons: rs12565727 (chr1); rs7349332 (chr2); rs4679955 (chr3); rs929626 (chr5); rs9668810 (chr12) and rs10502861 (chr18), as summarized in Fig 2A. This enhanced model explained 35% of the total variance of the risk of AGA with an overall significance of P = 5.286x10^-7^. Noticeable improvement of all accuracy parameters was achieved with the enhanced prediction model (Table 3). For the testing set including only the first two phenotype categories, AUC increased to 0.864. The overall level of correct predictions was higher at 74% (50% probability threshold) and 88.5% (65% threshold), with 39% inconclusive results. The number of correct predictions of bald phenotype in *phenotype category 1* increased from 66% to 76% compared to the simple 5-SNP model. An increase in the number of predictions was also noted for non-bald phenotype in *phenotype category 2* from 66% to 72%. Applying the 65% probability threshold, the level of correct predictions of bald stage reached the comparatively high level of 87.1% (27/31) in *phenotype category 1*. Moreover, the level of correct predictions of non-bald *category 2* reached 90% (27/30), substantially higher than the simple 5-SNP prediction model (71%). In similar results to the 5-SNP model, the enhanced prediction model did not work well with the general population sample of men at all ages and levels of balding. AUC for the testing set reflecting the full European population, although slightly better than the simple prediction model, was still low with an AUC of 0.66. Applying 65% probability threshold very weak prediction accuracy of non-bald *phenotype category 3* was found, with 42.4% correct predictions. Also, 67.7% of correct predictions of bald *phenotype category 4* were only slightly better than the 5-SNP model (Table 3). Finally, based on the results using the 20-SNP prediction model and 65% probability threshold, we investigated parameters of MPB prediction in the separated age groups: < 50 years (*phenotype categories 1* and *3*) and ≥ 50 years (*phenotype categories 2* and *4*). Overall accuracy of MPB prediction in men less than 50, expressed by AUC, was 0.657. This weak result was caused by very low specificity of prediction (42.4%, 28/66) while sensitivity was 87.1% (27/31). Values of PPV and NPV were 41.5% and 87.5%, respectively and there were 34.5% (51/148) inconclusive results. The overall accuracy of MPB prediction in men ≥ 50 years was markedly higher (AUC = 0.761). In this age group sensitivity was 67.7% (44/65) and specificity was relatively high at 90% (27/30). PPV and NPV were 56.3% and 93.6%, respectively (Fig 2B).

**Table 3:** Parameters describing the accuracy of prediction of MPB using 20-SNP logistic regression prediction model.

The enhanced 20-SNP model for male pattern baldness prediction				
Type of testing set samples	Phenotype categories 1 and 2		Phenotype categories 1, 2, 3 and 4	
Type of model	50% probability threshold	65% probability threshold	50% probability threshold	65% probability threshold
AUC	0.864		0.660	
Overall number of correct predictions %	74% (74/100)	88.52% (54/61)	59.06% (176/298)	65.63% (126/192)
Correct predictions of bald phenotype in *phenotype category 1*	76% (38/50)	87.10% (27/31)	76% (38/50)	87.10% (27/31)
Correct predictions of non-bald phenotype in *phenotype category 2*	72% (36/50)	90% (27/30)	72% (36/50)	90% (27/30)
Correct predictions of non-bald phenotype in *phenotype category 3*	-	-	48.98% (48/98*)	42.42% (28/66)
Correct predictions of bald phenotype in *phenotype category 4*	-	-	55.10% (54/98*)	67.69% (44/65)
Inconclusive results (non-prediction rate)	-	39% (39/100)	-	35.14% (104/296*)

^*^Four samples from phenotype categories 3 and 4 lacked data in one of the following SNPs: rs1160312, rs6625150, rs4679955 and therefore were excluded from the analyses.

**Fig 3:**
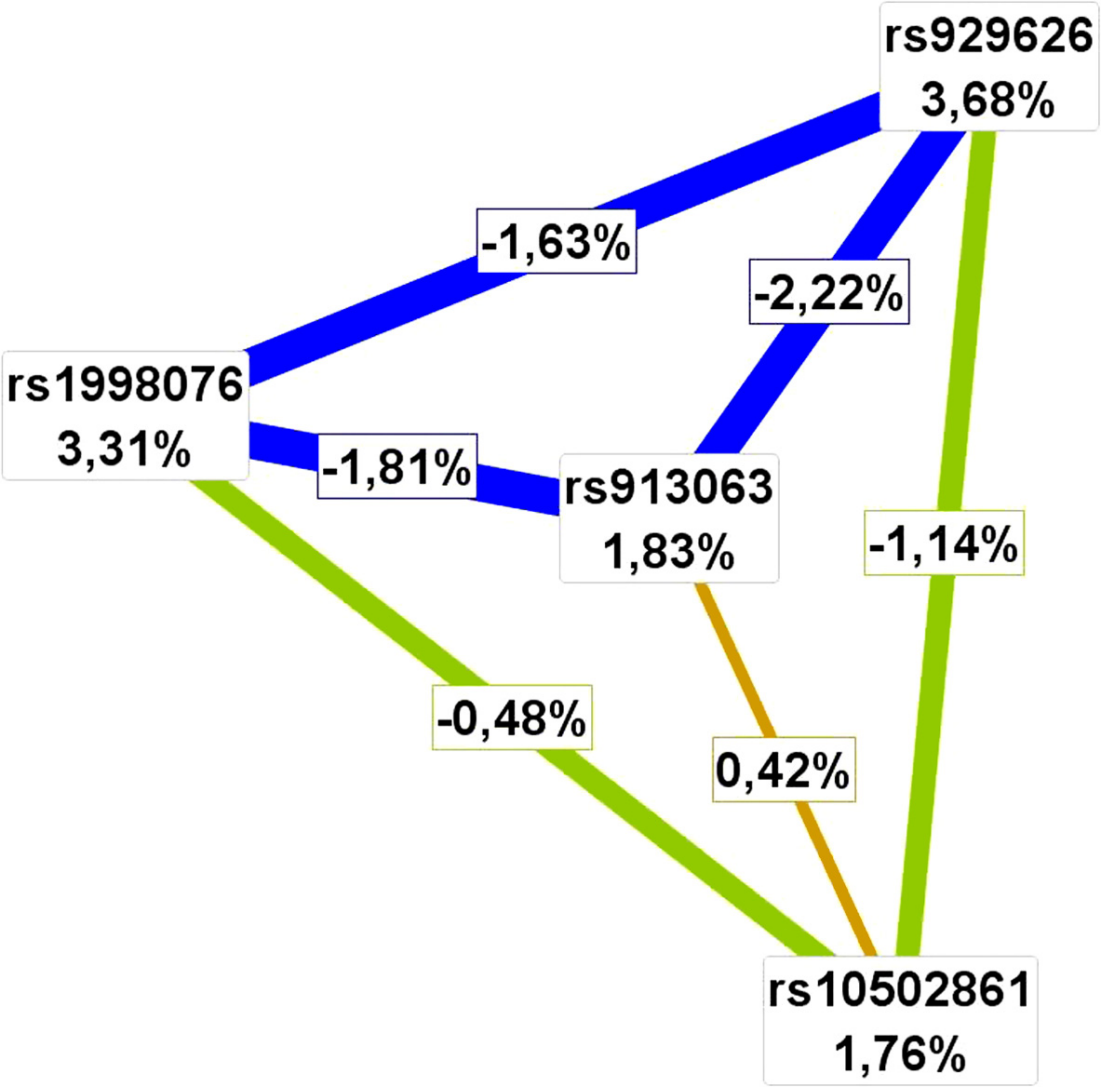
Entropy-based interaction graph from MDR analysis. Entropy values in the cells of individual SNPs indicate the main independent effects. Entropy values marked on the lines connecting two SNPs represent the entropy of interaction. Blue lines indicate a high degree of redundancy, green lines a reduced degree of redundancy and gold lines represent independence or additivity.

MDR was used to assess the possible effect of epistasis in explaining AGA variance and whether it improved prediction accuracy. MDR revealed high significance for a 3-factor model consisting of rs929626 (chr5), rs1998076 (chr20) and rs10502861 (chr18), taking into account the interaction between rs929626 and rs1998076 (P<0.001). According to the entropy-based analysis, this interaction has redundant character, (i.e. redundant information from both factors). SNP rs929626 (chr5) treated independently explains 3.68% of entropy, (i.e. it removes 3.68% of ‘uncertainty’ in AGA determination). SNP rs1998076 (chr20) treated as a single factor removes 3.31% of uncertainty. The entropy of the interaction between these two SNPs was -1.63%, suggesting this part of variation in determination of AGA is common for both SNPs (Fig 3). Importantly, this effect was confirmed by logistic regression (OR = 0.579, 95% CI = 0.448–0.747, P<0.001). Logistic regression analysis also indicated several additional interaction effects of statistical significance in the determination of AGA (data not shown) that suggest several epistatic effects for this trait, but this needs to be examined with a larger sample. The interaction between rs929626 and rs1998076 that both show independent main effects and are included in both prediction models, was found to have an ambiguous effect on prediction accuracy. It increased AUC from 0.864 to 0.870 when samples from *phenotype categories 1* and *2* were subjected to testing, but decreased AUC from 0.660 to 0.658 when the entire testing set was studied. Thus, the interaction was not included in the final 20-SNP prediction model.

## Discussion

Male pattern baldness is a type of androgenetic alopecia that affects susceptible individuals and progresses with age to form a key human externally visible trait. In European populations, the progressive loss of scalp hair can start at an early stage in adulthood. Rhodes et al. observed 16% of significant hair loss in men aged 18–29 years, increasing to 43–50% before the age of 50 [39].

The polygenic nature of MPB was discovered in early studies [40,41] and confirmed in GWAS analyses that followed [9,12,14,16]. The role of androgens had also been established as an important part of the predisposition to MPB and its age-related onset. Therefore, the androgenetic pathway is a process certain to contribute to MPB variation [27,42]. The Xq12 region that includes androgen receptor (*AR*, OMIM: 313700) continues to show the best-documented and strongest association with MPB. This region also contains ectodysplasin A2 receptor (*EDA2R*, OMIM: 300276), but it is still not fully understood whether one or both genes play a role in the progression of AGA. Our study confirmed that the *AR/EDA2R* region forms the major risk factor for MPB with the highest significance shown by rs5919324 located upstream of *AR*. The same SNP was previously shown to have the most significant association with MPB in the study of Cobb et al, 2010 [11]. Cobb found the second strongest association with MPB from the 20p11 region between *PAX1* encoding paired box protein 1 and *FOXA2* encoding forkhead box protein A2 [9,14]. From seven SNPs located in this region and included in our study, the highest significance was recorded for rs1998076, which was previously shown to be associated with MPB in a German population sample [14]. Richards et al. reported that the risk of AGA increases substantially (OR = 7.12) in individuals having at least one risk allele at both the Xq12 and 20p11 loci. They further estimated that these two loci have very high sensitivity of MPB prediction (98.2%) but very low specificity (6.6%) [9]. Interestingly, different SNPs on Xq12 have been shown to be most significantly associated in various European populations, indicating a complex genetic architecture for the region [4,8,10,11]. Moreover, the AGA-associated region on 20p11 contains no recognized genes to date, so it can only be speculated whether variation in this area has a regulatory function for *PAX1* and/or *FOXA2* located at each of the region’s ends [43]. It is also worth emphasizing that in our study six SNPs from Xq12 and five SNPs from 20p11 were found to have positive influence on prediction accuracy and thus all were included in the extended 20-SNPs prediction model. Further studies are necessary to analyze in more detail whether multiple DNA variants may contribute to MPB development in these two major AGA loci. If this is the case, it will have a strong impact on prediction modeling. Allelic heterogeneity is already recognized as an important factor affecting trait prediction analyses [21,25,37,44].

Beyond Xq12 and 20p11, ten additional loci have been shown to contribute to the overall risk of developing MPB [12,15,16]. From these, the most significant associations with AGA in our study were found for SNPs located on chromosomes 1, 5 and 7. Beyond rs5919324 (Xq12) and rs1998076 (chr20), three additional SNPs indicated independent effects when assessed with multivariate regression analysis. First, we found strong association for rs929626 located in an intron of *EBF1* (*early B-cell factor 1*), with this SNP originally identified in large meta-analysis of European males [16]. Transcription factor *EBF-1* encoded by *EBF1* is an important regulator of cell differentiation of adipocytes [45]; known to be involved in the regulation of hair follicle cycling [46]. SNP rs929626 was observed to have a positive impact on prediction in this study as well as interacting with rs1998076 (chr20). This interaction is novel and further studies on larger sample sets are necessary to confirm the effect and its biological mechanism. Implementation of the epistatic effect into the prediction model was found to have an ambiguous effect on accuracy and therefore was not included in the final 20-SNP prediction model. Second, rs12565727 close to *TARDBP* (*TAR DNA binding protein*) on chr1 gave the next highest association. This AGA determinant gene was recently identified by a meta-analysis performed by Li et al, in 2012. *TARDBP* codes for the 43 kDa transactive response DNA binding protein (TDP-43) and is involved in regulating gene expression and RNA splicing [47]. Third, the last SNP selected in our multivariate regression model was rs756853 located in an intron of *HDAC9 (histone deacetylase 9)* on chr7. This association was first discovered in a GWAS of German and Australian subjects [15]. The histone deacetylase 9 protein encoded by *HDAC9* belongs to the extensive HDAC superfamily, which plays a critical role in the regulation of gene expression. HDAC proteins are responsible for deacetylation of histones leading to chromatin condensation and consequently to transcriptional repression [48]. Brockschmidt, et al. suggested that *HDAC9* might interact with *AR* through MEF2C transcription factor modulating *AR* transcriptional activity [15]. Of six SNPs previously associated with hair morphology [30], none revealed independent association with MPB in our study. Only DNA variant rs7349332 in *WNT10A* showed a positive effect on prediction accuracy (a marginal AUC increase from 0.862 to 0.864) and was among markers included in the final 20-SNPs prediction model. This SNP was initially associated with hair morphology [30] and then also associated with MPB in a study of Heilmann et al. who suggested a possible link between hair curliness and hair loss [16].

Our results are in agreement with the genetic data obtained for AGA so far, indicating the involvement of multiple genetic loci with average or small individual effects. The study data indicates individuals carrying seven or more AGA risk alleles, in the five most associated loci, are significantly more susceptible to MPB. The genotype risk score calculated from the combined weighted number of risk alleles for these loci shows males with the highest genotype scores contrast to those with the lowest scores by having a three-fold increased MPB risk. These results can be compared with genotype risk score analysis made in the meta-analysis of Li et al. 2012 assessing 8 loci in more than 12,000 individuals [12]. Li’s study obtained OR = 5.78 but the genotype risk score calculated in our sample for the same variants gave a lower OR = 2.64. This is not unexpected, as the 8 SNPs lack associations with MPB in our samples and the gene from chr17 was not included in the 20-SNP model. In line with these findings, the regression model Li developed for the 8 SNPs was less accurate than our models (AUC = 0.742, data not shown). This could be explained by allelic heterogeneity and/or the small number of samples in our study. Overall, sample size was the main limitation of our study and this affects detection of small effect size variants and can prevent confirmation of weak association signals discovered in larger studies. We estimated that our sample size was sufficient to detect true effects with OR≤2 with 80% probability for only 18 of 50 tested DNA variants.

In other studies investigating susceptibility loci for AGA, selection of risk alleles/genotypes involved analysis of the two most contrasted phenotypic groups defined by age and MPB grade using a 50 year age division. Using these two phenotype groups for initial prediction model development, our study obtained an overall correct prediction rate of 66% for 5-SNP analyses using a 50% probability threshold, rising to 75.8% with the 65% threshold but with a 38% non-prediction rate for probabilities below the threshold. When extended to a 20-SNP prediction model, the overall number of correct predictions increased markedly to 88.5% (65% threshold, 39% non-prediction rate) with the 5-SNP AUC = 0.762 rising to AUC = 0.864 for the 20-SNP model. These results underline the observation that weaker predictors can give an important contribution to overall prediction accuracy when added to the strongest predictors identified [21]. Positive predictions reached 87.1% for *phenotype category 1* and 90% for *phenotype category 2* and these results can be considered to represent a high level of predictive performance. The highest level of correct predictions was recorded for males with Norwood-Hamilton V-VII grades of baldness (79.1%), whereas this only reached 56% for males with I-II grades of baldness (data not shown). In comparison with established forensic EVC prediction system such as IrisPlex [37], the predictive accuracy achieved in the two extreme MPB phenotype/age categories is lower than for blue/brown eye colour (AUC>0.9) and for red hair colour (AUC>0.9). However, it is similar to that reported for black, blond and brown hair colours (AUC>0.8, AUC = 0.81 and AUC = 0.82, respectively) and intermediate eye colours (AUC∼0.69–0.82) [21,37,49,50]. Good predictive performance has also been achieved for skin colour with only 1–3% prediction errors but relatively high 19–25% non-prediction rates [51,52]. Additionally, a recent forensic skin colour predictive test was developed showing successful prediction of white and black skin phenotype (AUC = 0.999 and AUC = 0.966, respectively) but slightly lower for intermediate skin phenotype (AUC = 0.803) [53]. It is worth noting that division of samples into four phenotype/age categories allows more insight into prediction accuracy observed in each of these subsets. The data clearly shows that, in contrast to *phenotype categories 1* and *2*, men <50 years without baldness of *phenotype category 3* give a much lower 42.4% correct predictions. The mean age in this sample subset was 32 years and it is possible that lower predictive performance reflects the insufficient time for MPB to develop in many of these subjects. Lower prediction accuracy was also obtained in *phenotype category 4* of men ≥50 years with significant baldness, with 67.7% correct predictions. It can be suggested that MPB is a heterogeneous phenotype and early onset and later onset alopecia could have different genetic backgrounds. This idea is indirectly supported by detailed analysis of AGA phenotypes in Europeans and Asians, discussed below. Furthermore, there are some indications that senescent alopecia has a distinct molecular etiology that increases the complexity of prediction of hair distribution in males [54,55]. Our study shows that the low specificity of 42.4% restricts MPB prediction in younger men and this observation is worth further investigation. MPB is a progressive condition and aging in males is accompanied by an increased incidence and degree of alopecia [27]. Therefore the differences between age-related scalp hair loss and early onset MPB will influence the applicability of a forensic hair distribution predictive test. For this reason, the ability to predict the biological age of a sample’s unknown donor will be an important adjunct to MPB predictive tests in forensic application. Considerable recent progress has been achieved in human age prediction [22–24,55] and further development of forensic MPB predictive tests will benefit from parallel studies in DNA-based age estimation. When we investigated two groups of men, aged <50 years and ≥50 years, better prediction parameters were obtained for men aged ≥50 years with sensitivity of 67.7% and specificity of 90%; meaning that in this age category the currently available set of genetic predictors provides a reasonable accuracy for application in forensic analyses.

Another aspect of MPB that needs detailed study concerns inter-population differences in AGA prevalence and molecular etiology that could influence the heterogeneity of the AGA phenotype. It has been suggested that the lowest incidence rate of MPB is observed in Africans [56]. Furthermore, AGA in Asians shows different characteristics compared to Europeans. In general, Japanese men develop MPB approximately 10 years later than European men and MPB is very rare in Japanese men less than 40 years old [57]. From the two most associated regions, only 20p11 has been shown to be associated with MPB in Asian subjects, while no Xq12 association has been detected [13,58]. Since the AR/EDA2R region contributes the most to the predictive performance of the SNPs we examined in European men, it is likely the prediction models developed in this study will not be applicable to analysis of Asian males. This aspect of the genetic control of AGA needs further investigation and indicates that forensic MPB prediction tests will benefit from tests for the inference of biogeographic ancestry [59]. Overall, the results obtained in this study clearly indicate MPB prediction in forensic DNA analysis is a viable option but should be applied with caution due to the variation in predictive accuracy relating to age and likely further variation from differences amongst populations.

## Conclusions

The results obtained from this study provide additional evidence that Xq12, comprising *AR/EDA2R* and the region on 20p11 are major determinants of AGA in European populations. Multiple DNA variants in these two regions have a major positive influence on the performance of tests designed to predict male pattern baldness. The three additional genes of *EBF1*, *TARDBP* and *HDAC9* showed weaker association with MPB but achieved statistical significance. The combination of 20 SNPs from ten known MPB-associated loci enabled the development of a test with good predictive performance for male pattern baldness in Europeans aged 50 years and older. Further studies are necessary to explain the causes of low specificity of prediction in males less than 50 years old, but insufficient time to develop MPB phenotypes in this age group is a plausible explanation. The role of senescent alopecia will also need to be addressed in future developments of predictive tests. However, the prediction models outlined here have a direct practical application in forensic analysis, particularly when combined with DNA tests for age and ancestry.

### Contributors

**Members of the EUROFORGEN-NoE consortium (European Forensic Genetics Network of Excellence) are:** University of Cologne: Peter M. Schneider (project coordinator, e-mail: peter.schneider@uk-koeln.de), Magdalena Bogus, Iva Gomes, Miriam Sirker, Theresa Gross; University of Santiago de Compostela: Ángel Carracedo (local coordinator), Victoria Lareu Huidobro, Christopher Phillips, Antonio Salas Ellacuriaga, Ana Mosquera Miguel, Vanesa Alvarez Iglesias, Manuel Fondevila; Norwegian Institute of Public Health: Peter Gill (local coordinator), Oskar Hansson, Øyvind Bleka; University of Copenhagen: Niels Morling (local coordinator), Claus Boersting; Netherlands Forensic Institute: Titja Sijen (local coordinator), Ate Kloosterman, Laurens Grol, Margreet van den Berge, Saskia Verheij; Innsbruck Medical University: Walther Parson (local coordinator), Mayra Mayr-Eduardoff, Christina Strobl; Norwegian University of Life Science: Thore Egeland (local coordinator), Guro Dørum; Northumbria University Centre for Forensic Science: Robin Williams (local coordinator), Martin Evison, Matthias Wienroth; Jagiellonian University: Wojciech Branicki (local coordinator), Józefa Styrna, Tomasz Kupiec, Ewelina Pośpiech; King’s College London: Denise Syndercombe-Court (local coordinator), David Ballard, Athina Vidaki; Epiontis GmbH: Sven Olek (local coordinator), Udo Baron, Helge Riemer.

## Supporting Information

S1 Fig Assessment of linkage disequilibrium between sets of SNPs in six regions using Haploview v4.2 software. LD color schemes represent values of r^2^ parameter between particular pairs of SNPs with white color indicating r^2^ = 0, shades of grey representing values of r^2^ between 0–1 and black color indicating complete linkage disequilibrium with r^2^ = 1. Values of r^2^ in squares are given in percentages.
(DOCX)

S1 Table Phenotypic description of the Norwood-Hamilton baldness categories. (DOCX)

S2 Table Characteristics of samples from the discovery set. (DOCX)

S3 Table Characteristics of samples included in the testing set. (DOCX)

S4 Table Genomic data and results of univariate association testing for the SNPs analyzed. (XLS)

S5 Table PCR and SBE primers used in the study. (DOCX)

S6 Table Discovery set genotypes. (XLS)

S7 Table Testing set genotypes. (XLS)

S8 Table The results of Hardy-Weinberg equilibrium analysis. (DOCX)

S9 Table Parameters describing the accuracy of prediction of MPB using the 5-SNP logistic regression prediction model. The 5-SNP model comprised: rs5919324, rs1998076, rs929626, rs12565727 and rs756853.
(DOCX)
