# Supplementary material for: Evaluation of DNA Variants Associated with Androgenetic Alopecia and Their Potential to Predict Male Pattern Baldness
Source: PLoS One. 2015 May 22;10(5):e0127852. doi: 10.1371/journal.pone.0127852 (PMC4441445; doi:10.1371/journal.pone.0127852)
Supplement: S1 Fig — LD color schemes represent values of r2 parameter between particular pairs of SNPs with white color indicating r2 = 0, shades of grey representing values of r2 between 0–1 and black color indicating complete linkage disequilibrium with r2 = 1. Values of r2 in squares are given in percentages. (DOCX) [file pone.0127852.s001.docx]

**S1 Fig. Assessment of linkage disequilibrium between sets of SNPs in six regions using Haploview v4.2 software.** LD color schemes represent values of r^2^ parameter between particular pairs of SNPs with white color indicating r^2^=0, shades of grey representing values of r^2^ between 0-1 and black color indicating complete linkage disequilibrium with r^2^=1. Values of r^2^ in squares are given in percentages.

SNPs on chromosome 1

**
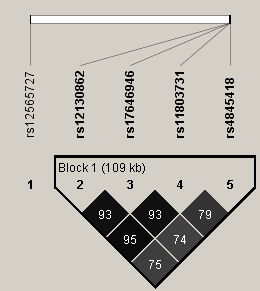
**

SNPs on chromosome 2

**
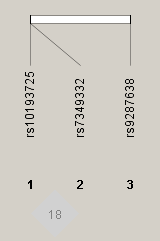
**

SNPs on chromosome 7

**
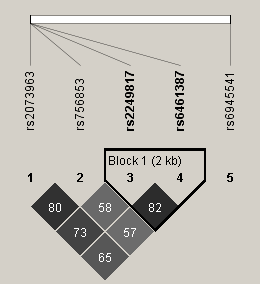
**

SNPs on chromosome 17

**
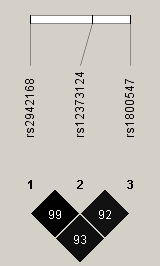
**

SNPs on chromosome 20

**
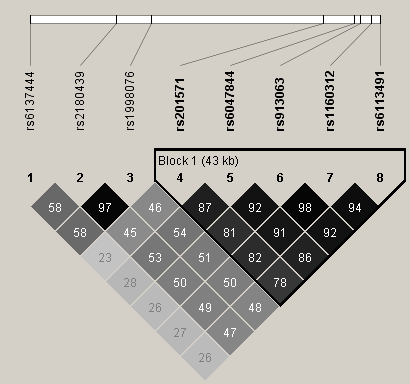
**

SNPs on chromosome X

**
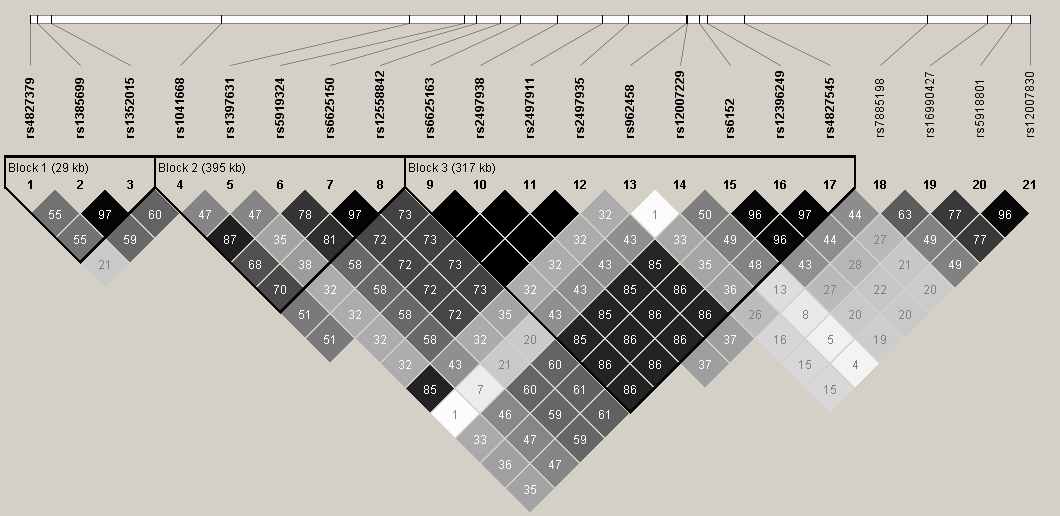
**
