# Supplementary material for: Evaluation of DNA Variants Associated with Androgenetic Alopecia and Their Potential to Predict Male Pattern Baldness
Source: PLoS One. 2015 May 22;10(5):e0127852. doi: 10.1371/journal.pone.0127852 (PMC4441445; doi:10.1371/journal.pone.0127852)
Supplement: S1 Table — (DOCX) [file pone.0127852.s002.docx]

**S1 Table. Simplified phenotypic description of the Norwood-Hamilton baldness categories.**

| **The Norwood-Hamilton scale of male-pattern baldness** | **Phenotypic description** |
| --- | --- |
| Grade I | no recession of the hair line |
| Grade II | minor recession of the frontal hairline |
| Grade III | deep symmetrical recession at the temples |
| Grade III vertex | significant frontal hair loss coupled with hair loss at the crown of the head |
| Grade IV | deepening frontal recession in the temples and progressively more hair loss at the crown |
| Grade V | hair loss at the vertex and front temporal areas are extended |
| Grade VI | the frontal and vertex regions of hair loss merge into one area and increase in size |
| Grade VII | the most advanced stage of male-pattern baldness, in which all hair is lost along the front hairline and crown |
