# Supplementary material for: Evaluation of DNA Variants Associated with Androgenetic Alopecia and Their Potential to Predict Male Pattern Baldness
Source: PLoS One. 2015 May 22;10(5):e0127852. doi: 10.1371/journal.pone.0127852 (PMC4441445; doi:10.1371/journal.pone.0127852)
Supplement: S2 Table — (DOCX) [file pone.0127852.s003.docx]

**S2 Table. Characteristics of samples from the discovery set**.

| Discovery set | Grade of baldness | Poland | Netherlands | England | Spain-Italy | All |
| --- | --- | --- | --- | --- | --- | --- |
| Phenotype category 1 | III | 48 | 16 | 10 | 8 | 82 (26.89%) |
|  | IV | 28 | 3 | 2 | 3 | 36 (11.80%) |
|  | V | 17 | 3 | 1 | 1 | 22 (7.21%) |
|  | VI | 20 | 8 | 3 | 2 | 33 (10.82%) |
|  | VII | 3 | 0 | 0 | 0 | 3 (0.99%) |
| Phenotype category 2 | I | 32 | 5 | 4 | 1 | 42 (13.77%) |
|  | II | 76 | 3 | 2 | 6 | 87 (28.52%) |
| All | All | 224 (73.4%) | 38 (12.5%) | 22 (7.2%) | 21 (6.9%) | **305 (100%)** |
