# Supplementary material for: Evaluation of DNA Variants Associated with Androgenetic Alopecia and Their Potential to Predict Male Pattern Baldness
Source: PLoS One. 2015 May 22;10(5):e0127852. doi: 10.1371/journal.pone.0127852 (PMC4441445; doi:10.1371/journal.pone.0127852)
Supplement: S3 Table — (DOCX) [file pone.0127852.s004.docx]

**S3 Table. Characteristics of samples included in the testing set**.

| Stage  of baldness | Age | Phenotype category | Poland | Netherlands | England | Spain  -Italy | Denmark | Germany | All |
| --- | --- | --- | --- | --- | --- | --- | --- | --- | --- |
| Bald | <50 | 1 | 22 | 6 | 4 | 3 | 7 | 8 | 50  (16.67%) |
| Non-bald | ≥50 | 2 | 37 | 3 | 2 | 3 | 3 | 2 | 50  (16.67%) |
| Non-bald | <50 | 3 | 69 | 2 | 6 | 5 | 15 | 3 | 100  (33.33%) |
| Bald | ≥50 | 4 | 96 | 0 | 2 | 0 | 2 | 0 | 100  (33.33%) |
| All | | | 224  (74.7%) | 11  (3.7%) | 14  (4.7%) | 11  (3.7%) | 27  (9.0%) | 13  (4.3%) | 300  (100%) |
