# Supplementary material for: Evaluation of DNA Variants Associated with Androgenetic Alopecia and Their Potential to Predict Male Pattern Baldness
Source: PLoS One. 2015 May 22;10(5):e0127852. doi: 10.1371/journal.pone.0127852 (PMC4441445; doi:10.1371/journal.pone.0127852)
Supplement: S5 Table — (DOCX) [file pone.0127852.s006.docx]

**S5 Table. PCR and SBE primers used in the study**.

|  | **SNP** | **PCR primers** | **Conc. [μl]**  **PCR primer** | **Extension primer** | **Conc. [μl]**  **SNP primer** |
| --- | --- | --- | --- | --- | --- |
| **Multiplex 1** | | | | | |
| 1 | rs11803731 | F: TCCGGGAGAAACCGTTGTT  R: TCAGAGGCTGTCGGCCAGG | 0.125  0.125 | ctctctctctCCCGCTGCTCCCGCA | 0.3 |
| 2 | rs17646946 | F: CCCAGAGTACCAGCTATTTC  R: GCTCCAGAGCAGGAAGATAT | 0.25  0.25 | tctctctctctctctGCCAGGAACTGGAGT | 0.7 |
| 3 | rs756853 | F: TTATGAGGCACAGACCCTTG  R: CCCTACGCTTTCATTTTTGG | 0.125  0.125 | tctctctctctctGTCACACTTCTGCCT | 0.5 |
| 4 | rs1268789 | F: TTCCCAGCTCCATCATATGC  R: AAATCAGTCAACCAACCTTG | 0.125  0.125 | ctctctctctctctctctctCATATGCAATCCCCCA | 0.4 |
| 5 | rs2497938 | F: ATCCTGAATACCAAGCCCTC  R: CCCAGCTTTGCCATTTGTAG | 0.25  0.25 | tctctctctctctctctAATGGTAAAGCACTGTAAG | 0.7 |
| 6 | rs6625163 | F: GTCATCGAACCCTGTTTCAC  R: GCAGGATGATTTTCAAAGCG | 0.125  0.125 | tctctctctctctctctctctctGCCCACAACACAAGACC | 0.1 |
| 7 | rs6047844 | F: ATAACGATGGCAATGGGAAG  R: CTTTGGACAAGGGACCACAC | 0.3125  0.3125 | ctctctctctctctctctAAGTCAGGATAGCCAGTATAG | 1.7 |
| 8 | rs5918801 | F: CACGGACCTGGAGGCTATTA  R: GCACCCAACAGGTGACTTTT | 0.3125  0.3125 | ctctctctctctctctctctctctctTCAGTGTTCATTACCCCA | 1.1 |
| 9 | rs2073963 | F: CTTGGAATTCAGAGCTGTTG  R: TTATCTGCTCTGAGCGCTTC | 0.3125  0.3125 | tctctctctctctctctctctctctTGTTACTTTCAGGGATCA | 0.9 |
| 10 | rs6461387 | F: GCTAATCCAAGTGACAACCC  R: TCCATGGTGACATGATAGAG | 0.3125  0.3125 | ctctctctctctctctctctctctctctGACAACCCATAGTCTAACTA | 1.0 |
| 11 | rs913063 | F: TGCTGAGGAATGCCACATCT  R: GAGGAAGAAATGCCCCATCT | 0.25  0.25 | ctctctctctctctctctctctctctctctctctCTCTCTCTTCTGTTTGCC | 1.1 |
| 12 | rs12007830 | F: GTAACTAATGCCAACACACC  R: ACCAGAGTTCAGCTATTTGC | 0.125  0.125 | ctctctctctctctctctctctctctctctctctctGTTCAGCTATTTGCAGTGAT | 0.6 |
| **Multiplex 2** | | | | | |
| 13 | rs2249817 | F: TACGGCATCATAGGTTCACG  R: GCCATGAGTGGTGGAGAGAT | 0.375  0.375 | TGTTCATTATAGTGACCAAAA | 1.3 |
| 14 | rs6945541 | F: GGTTTGGTTGTAATTTGAATGGA  R: CCTGAACCTAGGCAGAACCAA | 0.125  0.125 | CAGAACCAATTCCAAGTATC | 0.5 |
| 15 | rs6137444 | F: GCAGAAACAACATAATTGCC  R: CTTGGCCCTGGCTACATTTG | 0.25  0.25 | TGGATTAGACTGCTAACTTTTTAAA | 0.8 |
| 16 | rs7349332 | F: ATCCCAGCCCATACCAACG  R: AACAGCTCCAGAACATCTGC | 0.125  0.125 | tctctctctctctCAGAACATCTGCTTCCCAC | 0.4 |
| 17 | rs10502861 | F: CTAGGAAGTACCCATTCCTG  R: TCCAGGGGCATTTTTCCAAC | 0.1875  0.1875 | ctctctctctctctCCCATTCCTGATAGTAAGCAAG | 0.5 |
| 18 | rs201571 | F: CGAGCCACTATCCTTTGAGC  R: AGCCACCCTGAACTTTGTGA | 0.0625  0.0625 | ctctctCATCACTCTTGTCCATTCAAATTTTTTCTG | 0.4 |
| 19 | rs12558842 | F: TTCTTTAAAGGCCACCCAGC  R: CATAGTTTCTTCTCATGAAC | 0.375  0.375 | tctctctctctctctCTTCTCATGAACTTTTTTCTCGCAGGA | 0.5 |
| 20 | rs1160312 | F: TCAGGGCTTGCTGGACTCTC  R: ACTCTCTGCTATCAGCATCC | 0.0375  0.0375 | tctctctctctctctctctTCCTGGCAGTGTGGGGTCAGGACTC | 0.05 |
| 21 | rs6113491 | F: CTCACCCTAAAAGGAAAACC  R: AAAATTGGTGGTGCCAGAGG | 0.1875  0.1875 | ctctctctctctctctctctctctctAGGAAAACCTGTACCCATTAAG | 0.8 |
| 22 | rs6625150 | F: CACAGTGATGGAAAGAATTTTGA  R: CCTGATATAAGAGGGCTCAGTAAA | 0.25  0.25 | ctctctctctctctctctctctctctCCTTATTCACTGCTCTTAGAAC | 1.0 |
| 23 | rs5919324 | F: AAAGGGCCCATCTCAAAAAT  R: GCGTGGGATTAGATAAAAATGG | 0.0625  0.0625 | tctctctctctctctctctctctctctTACAGAAGACATCCCCCAGGGCTTGGG | 0.4 |
| 24 | rs4845418 | F: CCTGGAGAGGAGCACAACTAA  R: GCGTCACCTTCAGAAACCAC | 0.0625  0.0625 | ctctctctctctctctctctctctctctACTGCCCTGTTAATTATAGGCCATTTC | 0.2 |
| **Multiplex 3** | | | | | |
| 25 | rs12130862 | F: AACTTTGTGGATCTCATACC  R: AAGGAAGAGGCAAAATGGTC | 0.1875  0.1875 | ATTTCTACCTAGGGCATC | 0.5 |
| 26 | rs16990427 | F: CCTCTGCATGTATTTCCCTC  R: AGAGCCTACACACAAGATCC | 0.0625  0.0625 | tctctctctctTGGACTTCACAGGCT | 0.2 |
| 27 | rs12565727 | F: TCTGTTTACCTGGGGCATTG  R: ATTACCACATGGTCCAAGGC | 0.125  0.125 | tctctctctctCAAGGCCACCAGCAT | 0.5 |
| 28 | rs1397631 | F: CCAGTTGAAAAACATGGTAAG  R: GGGTCACATTCAGACAATTC | 0.0625  0.0625 | tttttttTCACATTCAGACAATTCAGGGACA | 0.2 |
| 29 | rs7885198 | F: GTCCTCTAATTATCCATACG  R: GGCATCAAAATGGACACTTC | 0.25  0.25 | ctctctctTAATTATCCATACGTTACATGCTGTTAA | 0.3 |
| 30 | rs962458 | F: TCTGGCTTTATTCCGTAGCC  R: TGGATGGGACCTAGCCATAG | 0.375  0.375 | tctctctctctctctctAGCCTTCTGGAAAACAT | 1.1 |
| 31 | rs1352015 | F: GCTTTTTCTTCCCCAGTTAG  R: AGCACTGATGATGAAGTAGC | 0.0625  0.0625 | ctctctctctGCACTGATGATGAAGTAGCAAACACAATA | 0.08 |
| 32 | rs1385699 | F: CCTTCTGAACACGATTGATG  R: CCTCCTCGCAGGTACAAAAG | 0.0625  0.0625 | ctctctctctctctctctctctctGCAGCTGGGGCCACCACA | 0.2 |
| 33 | rs2497935 | F: CAAGCATGCCTGGTAAACTG  R: AGTATCTGATCTGAGGAGGC | 0.25  0.25 | tctctctctctctctctctctctTTGATCCTCTGTCATATAAGC | 0.7 |
| 34 | rs1041668 | F: CCTCCCTTTACCTACTAGAC  R: TCACACTTAGGAGGTGCTAC | 0.25  0.25 | ctctctctctctctctctctctctctctACAACAGACATGGAACTACTT | 0.6 |
| 35 | rs12396249 | F: TGACCGTGTCTCAGACTAAG  R: TCCAGAATTCAGCCACTTCC | 0.0625  0.0625 | ctctctctctctctctctctCCGTGTCTCAGACTAAGGTGGTATTGGT | 0.07 |
| 36 | rs4827545 | F: GCCAGCTTAAGATGCTCTGG  R: AAACACTTCATTGCAGGTCTGA | 0.1875  0.1875 | tttttttttttttttttttttttCTGATTTATTATTAGGTTGGCCAATACA | 0.4 |
| 37 | rs1998076 | F: TGGCATCTCCAATAGGACAAA  R: GGTTGCCCAAATCAAGCTAA | 0.1875  0.1875 | tctctctctctctctctctctctctGTCTATACAACAGAAAAGATTTTGATTCT | 0.6 |
| 38 | rs12007229 | F: ATGCTGTGGTCTCATATGGC  R: CTTGGTGGAGGGAGTTCATC | 0.0625  0.0625 | tctctctctctctctctctctctctctctctGTGGAGGGAGTTCATCCCCCTTTCTT | 0.08 |
| 39 | rs2497911 | F: CTGTTGAGCGAGGTGGAAAT  R: ATTCAACTCCTCCCCCACAT | 0.0625  0.0625 | tctctctctctctctctctctctctctctctctACTCCTCCCCCACATTTTAACAACAGCT | 0.08 |
| **Multiplex 4** | | | | | |
| 40 | rs4827379 | F: TCGTCCTCCTCTAAGTTCTG  R: CTCTCTAGACCCTATGTAGC | 0.125  0.125 | GCCTAGAGAAAAGAGGGAGAGAAA | 0.15 |
| 41 | rs6152 | F: CCATGCAACTCCTTCAGCAA  R: GCGTTGTCAGAAATGGTCGA | 0.05  0.05 | AAGGCAGCAGCAGCGGGAGAGCGAGGGA | 0.05 |
| 42 | rs2180439 | F: TCCCATGAGCCAGTCTCTTC  R: GGGGCAGTGAAACCATTCTA | 0.125  0.125 | TGATGCTAGCTGCCGTTTTGTGTTAT | 0.2 |
| 43 | rs9287638 | F: GGCAAGTATGGACGGATGAA  R: GCTGCTCTGTAGCCTCATAAA | 0.3125  0.3125 | TTTTCCACAGTGAAAAAGGAAACAGCAGTTTTAAAC | 0.9 |
| 44 | rs12373124 | F: GGGTCGACATGCTCTTTCTTA  R: GAGCTGCCCTTCATCCC | 0.375  0.375 | ttttttttttttttttttCCTGGGCCTGGGCATGGAC | 1.5 |
| 45 | rs2942168 | F: AGAGCAGGGAGGGATGTAG  R: ACTAGGAAGGTATCCAGCCTTT | 0.125  0.125 | tttttttttttttttttTGTGGGGCTCTGGGAAAGAGCTT | 0.3 |
| 46 | rs1800547 | F: CGGTGGTTTCTAGATGTGACAG  R:ACAGGAGGATAAACACGCAAAG | 0.125  0.125 | tttttttttttttttttttttACTGCCTGGGGGTCTCTGGGGCT | 0.15 |
| 47 | rs10193725 | F: TTTCAAGGTGTGGGGATGG  R: CAGAGAAGGCAATGGAAGGA | 0.25  0.25 | ttttttttttttttttttttttttttttGGGGCAGCTTCAGGGAGGAA | 1.2 |
| 48 | rs929626 | F: AAGAAACGCATACCCAGGTG  R: CGTAACCTGCCAAAGCCTTA | 0.3125  0.3125 | ttttttttttttttttttttttttTCATAGGATTGTGAGACTTGGAGA | 1.3 |
| 49 | rs9668810 | F: AAGCCTCTCTCCTCTGCTTG  R: GGAGCCTTTGAAGGGGTAAT | 0.125  0.125 | tttttttttttttttttttttCCAAGGCGATTGGTGTCCTTATAAAGAGAGA | 0.15 |
| 50 | rs4679955 | F: AGTGGGAGTGACGATGGAGA  R: TGGTGAATGTGACCAACTTGA | 0.125  0.125 | tttttttttttttttttttttttttGTGAATGTGACCAACTTGAAATTACACGATC | 0.1 |
