# Supplementary material for: Evaluation of DNA Variants Associated with Androgenetic Alopecia and Their Potential to Predict Male Pattern Baldness
Source: PLoS One. 2015 May 22;10(5):e0127852. doi: 10.1371/journal.pone.0127852 (PMC4441445; doi:10.1371/journal.pone.0127852)
Supplement: S8 Table — (DOCX) [file pone.0127852.s009.docx]

**S8 Table. The results of Hardy-Weinberg equilibrium analysis**.

| Nr | SNP | Obs. Het | Exp. Het | P-value |
| --- | --- | --- | --- | --- |
| 1 | rs11803731 | 0.32884 | 0.35103 | 0.12040 |
| 2 | rs17646946 | 0.34907 | 0.36366 | 0.33950 |
| 3 | rs756853 | 0.46543 | 0.47600 | 0.55564 |
| 4 | rs1268789 | 0.44182 | 0.42316 | 0.37277 |
| 5 | rs6047844 | 0.46880 | 0.49062 | 0.29139 |
| 6 | rs2073963 | 0.43676 | 0.46667 | 0.20812 |
| 7 | rs6461387 | 0.46037 | 0.47637 | 0.44089 |
| 8 | rs913063 | 0.47218 | 0.49365 | 0.36574 |
| 9 | rs6945541 | 0.50422 | 0.49085 | 0.53485 |
| 10 | rs2249817 | 0.46880 | 0.49014 | 0.31644 |
| 11 | rs6137444 | 0.51265 | 0.47213 | 0.04109 |
| 12 | rs7349332 | 0.19056 | 0.20191 | 0.22842 |
| 13 | rs10502861 | 0.38111 | 0.37489 | 0.75218 |
| 14 | rs201571 | 0.47555 | 0.48160 | 0.77248 |
| 15 | rs1160312 | 0.47892 | 0.49365 | 0.50693 |
| 16 | rs6113491 | 0.48229 | 0.49612 | 0.47525 |
| 17 | rs4845418 | 0.29005 | 0.30665 | 0.22119 |
| 18 | rs12130862 | 0.33558 | 0.35652 | 0.15614 |
| 19 | rs12565727 | 0.34401 | 0.34451 | 1.00000 |
| 20 | rs1998076 | 0.48735 | 0.48785 | 1.00000 |
| 21 | rs2180439 | 0.48398 | 0.48675 | 0.94089 |
| 22 | rs9287638 | 0.47218 | 0.46073 | 0.64297 |
| 23 | rs12373124 | 0.25970 | 0.27613 | 0.19416 |
| 24 | rs2942168 | 0.25801 | 0.27500 | 0.14168 |
| 25 | rs1800547 | 0.23946 | 0.26237 | 0.04188 |
| 26 | rs10193725 | 0.32715 | 0.31291 | 0.31554 |
| 27 | rs929626 | 0.49073 | 0.49837 | 0.72030 |
| 28 | rs9668810 | 0.39966 | 0.38725 | 0.47465 |
| 29 | rs4679955 | 0.49747 | 0.49422 | 0.92683 |
