# Supplementary material for: Evaluation of DNA Variants Associated with Androgenetic Alopecia and Their Potential to Predict Male Pattern Baldness
Source: PLoS One. 2015 May 22;10(5):e0127852. doi: 10.1371/journal.pone.0127852 (PMC4441445; doi:10.1371/journal.pone.0127852)
Supplement: S9 Table — The 5-SNP model comprised: rs5919324, rs1998076, rs929626, rs12565727 and rs756853. (DOCX) [file pone.0127852.s010.docx]

**S9 Table.** **Parameters describing the accuracy of prediction of MPB using the 5-SNP logistic regression prediction model.**

The 5-SNP model comprised: rs5919324, rs1998076, rs929626, rs12565727 and rs756853.

| The simple 5-SNP model for male pattern baldness prediction | | | | |
| --- | --- | --- | --- | --- |
| Type of testing set samples | Phenotype categories 1 and 2 | | Phenotype categories 1, 2, 3 and 4 | |
| Type of model | 50% probability threshold | 65% probability  threshold | 50% probability threshold | 65% probability  threshold |
| AUC | 0.762 | | 0.594 | |
| Overall number of  correct predictions % | 66% (66/100) | 75.81% (47/62) | 54.67% (164/300) | 58.38% (108/185) |
| Correct predictions of bald phenotype in *phenotype category 1* | 66% (33/50) | 87.10% (27/31) | 66% (33/50) | 87.10% (27/31) |
| Correct predictions of non-bald phenotype in *phenotype category 2* | 66% (33/50) | 70.97% (20/31) | 66% (33/50) | 70.97% (20/31) |
| Correct predictions of non-bald phenotype in *phenotype category 3* | - | - | 46% (46/100) | 37.29% (22/59) |
| Correct predictions of bald phenotype in *phenotype category 4* | - | - | 52% (52/100) | 60.94% (39/64) |
| Inconclusive  results (non-prediction rate) | - | 38% (38/100) | - | 38.33% (115/300) |
